# Proteomic Analyses Unveil Actionable Disease Pathways in COVID-19: A Step Toward Targeted Therapies∗

**DOI:** 10.1016/j.jacbts.2022.03.011

**Published:** 2022-05-23

**Authors:** George Vasquez-Rios, Girish N. Nadkarni

**Affiliations:** a Barbara T. Murphy Division of Nephrology, Department of Medicine, Icahn School of Medicine at Mount Sinai, New York, New York, USA; b Mount Sinai Clinical Intelligence Center, Icahn School of Medicine at Mount Sinai, New York, New York, USA; c Division of Data-Driven and Digital Medicine, Icahn School of Medicine at Mount Sinai, New York, New York, USA

**Keywords:** COVID-19, genomics, myocarditis, sequela

**Figure:**
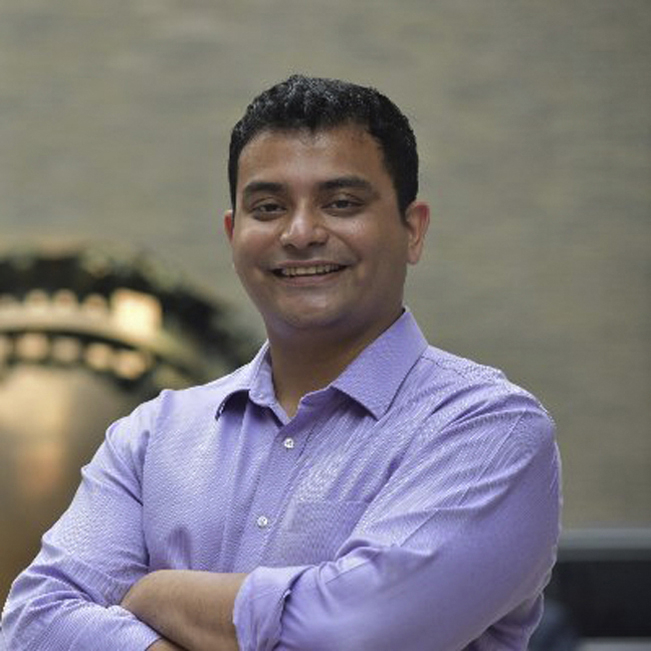

Therapies targeted to critical pathways implicated in the pathophysiology of COVID-19 caused by SARS-CoV-2 infection are desperately needed. Although the incidence of COVID-19 cases seems to have decreased in several regions, many patients remain vulnerable including older, immunocompromised, or unvaccinated individuals. To reduce the burden of COVID-19 in the acute phase and address postacute sequelae of SARS-CoV-2 infection, comprehensive analytical approaches are pivotal to uncover pathways amenable to translation into drug development.

In this issue of *JACC: Basic to Translational Science*, Roh et al^1^ conducted a matched case-control study examining the plasma proteome of 80 patients with different severities of COVID-19 disease, including those hospitalized (but not in intensive care units) and individuals requiring intensive care with different degrees of cardiac involvement along with non–COVID-19 controls. A series of unsupervised enriched principal component analyses revealed that protein profiles related to innate cellular inflammation, senescence, and RNA processing had the highest biological relevance and distinguished between those with and without COVID-19. Furthermore, these proteomic signatures defined different degrees of cardiac involvement. Thus, senescence-associated secretory phenotype (SASP)-1 and -2 emerged as important regulatory proteins in the primary and validation cohorts. Finally, transcriptional profiles in mouse models infected with SARS-CoV-2 were found to be enriched for both SASP-1 and SASP-2.

Senescence in several cell types is promoted by damage/danger signals as well as a myriad of metabolic, redox, and mechanical forces. Specific transcription factor cascades (ie, p16 or p21) induce extensive changes in gene expression, organelle dysfunction, and epigenomic remodeling. A subset of senescent cells develops a secretory profile, thereby releasing inflammatory cytokines, proteases, and profibrotic factors, which can affect neighboring and distant tissues. Many of the clinical conditions connected to cellular senescence share features with complications from COVID-19, including frailty/weakness, lung dysfunction/fibrosis, and lower cardiomyocyte reserve. Previous investigators have demonstrated that senolysis (targeted depletion of senescent cells) by drugs such as bafilomycin A1 (via inhibition of autophagy), fisetin/quercetin (flavonoids that inhibit the NLRP3 inflammasome), and navitoclax (used in combination with other agents to inhibit BCL-2 antiapoptotic family proteins) could potentially mitigate the toxic effects from SASP-1/SASP-2 in preclinical and clinical models. More recently, targeting virus-induced senescence cells with navitoclax and a combination of dasatinib plus quercetin has been found to selectively eliminate virus-induced senescence cells, protect against lung disease, and reduce tissue inflammation in COVID-19–infected hamsters and mice.^2^ Thus, SASP-1/SASP-2 pathways may offer an appealing target to reduce not only cardiovascular morbidity but other deleterious effects from COVID-19 and, perhaps, clinical sequela. However, SASP-1/SASP-2 signals may also play a role in beneficial processes such as the elimination of injured tissue, tumorigenesis regulation, and wound healing. Therefore, additional human and mechanistic studies are needed to understand this fine balance.

We point out some interesting observations in the paper. First, of 171 plasma proteins differentially expressed in COVID-19 patients with severe cardiac injury, REG3G (regenerating islet-derived protein 3 gamma) proteins were found to be the most abundant. Moreover, REG1a and REG1b proteins (c-type lectin superfamily), which are presumed to modulate cellular responses to infection in the gastrointestinal tract and lung, were also statistically associated with severity. Intriguingly, STAT3 (signal transducer activator 3), and JAK2 (Janus kinase 2)—key proteins that modulate *REG3G*—gene expression in response to a broad range of interleukins did not reach statistical significance. Although part of this finding could be related to reduced power from a small sample size, it is possible that this pathway is not selectively activated in patients predominantly suffering from cardiac injury in COVID-19. Conversely, activation of STAT3 after via MAPK signaling pathways (phosphorylating serine 727) has been found to be critical to induce kidney cell injury in patients with acute kidney injury and COVID-19.^3^ Interestingly, members of the MAPK family evaluated in the current study (MAPK1, MAPK8, and MAPK14) did not reach statistical significance either, suggesting the possibility that *REG3G* regulation may occur through alternative and, perhaps, disease-specific mechanisms. In light of the recent U.S. Food and Drug Administration approval of JAK inhibitors (baricitinib, ruxolitinib, and tofacitinib) for critically ill patients with COVID-19,^4^ careful investigations of the JAK-STAT/MAPK-STAT pathways are needed to identify suitable scenarios for their use.

Second, the inverse association between low ADAMTS13 levels with severe COVID-19 is important. A body of literature suggests the presence of microthrombi in the delicate circulatory network of the heart, kidneys, and lungs. However, multiple inflammatory and endothelial cell injury compounds could be responsible for triggering consumptive coagulopathy. Proteomic technologies may be sensitive to alterations in protein structure because of oligomerization, degradation, and post-translational modifications that can unpredictably alter protein binding affinity and quantification. These factors could affect the identification of a broader range of proteins involved in microthrombi formation and myocardial injury in COVID-19. Indeed, Roh et al^1^ found that ADAMTS13 had a significant yet weak negative association with cardiac troponins, a finding that was reproduced in the independent cohort. However, to overcome the limitations from residual confounding and reverse causation, Roh et al^1^ conducted an automated 2-sample mendelian randomization to genetically estimate ADAMTS13 protein levels using locus-wide significant Protein Quantitative Trait Loci (pQTLs). All locus-wide intronic cis-pQTLs that were significant were ranked by effect size and pruned for independence (*R*^2^ < 0.10). A series of analyses ultimately yielded 6 single-nucleotide variations (SNVs, formerly SNPs) that served as genetic proxies for ADAMTS13. These SNPs were deemed to be causally related to “other forms of acute ischemic heart disease”—a surrogate classification for myocardial injury used by Roh et al^1^.

The aforementioned results deserve careful attention because the reduction of ADAMTS13 gene expression (67% lower among the SARS-CoV-2 infected animals in the study) may not necessarily be associated with the full spectrum of Thrombotic Thrombocytopenic Purpura (TTP) in humans. In fact, thrombotic microangiopathies and acquired TTP have been infrequently seen in COVID-19. However, relatively low ADAMTS13 levels could still mediate part of the microvascular damage perceived in COVID-19 in the context of other severe physiologic stressors. Also, several SNVs in ADAMTS13 have been found to be causally related to cardiac heart disease and stroke in noninfected patients; reinforcing the need for additional studies on this coagulation pathway. Furthermore, this is particularly important because different degrees of bleeding diathesis and thrombotic abnormalities have been reported among COVID-19 patients even when only modest laboratory abnormalities are reported. Therefore, there could be a role for genetic testing to identify the patients who would be at the greatest risk for coagulation abnormalities and microvascular thrombosis and would potentially benefit from novel therapies such as recombinant human ADAMTS13.^5^

Although Roh et al^1^ present a comprehensive plasma proteomic analysis conducted in a small set of individuals during the first pandemic surge, following a matched case-control design that reveals novel clinical and preclinical data, it is important to notice some limitations. First, by design, Roh et al^1^ focused on strict inclusion criteria to identify individuals with evidence of cardiac injury via abnormal biomarkers. Therefore, it is unclear if a subgroup of patients underwent coronary interventions and were actually found to have myocardial infarction, affecting the internal validity of the study. Also, epidemiologic reports suggest that patients affected from COVID-19 during the first surge were sicker than in subsequent surges. Thus, it is possible that homeostatic alterations found in this study (ie, SASP-1/SASP-2 pathways) may not be reproduced to the same extent in today’s patient population. Moreover, it is intriguing whether the lack of statistical significance of important pathways such as JAK/STAT or JAK/MAPK is a reflection of a particular subphenotype of COVID-19—perhaps closely related to cardiac injury/stress or lack of statistical power. Additional studies including nonlinear modeling could serve to identify if these pathways (or associated genes) could still be differentially expressed in patients with a broader spectrum of complications. Moreover, this could serve to identify patients who would benefit the most from JAK inhibitors while limiting toxic side effects.

There has been an important progress in the pursuit of precision COVID-19 treatments over the past year. This study adds important information on actionable pathways that could serve to repurpose medications and investigate novel therapies. Unfortunately, crude treatment assignments based on clinical inclusion criteria rather than genetic and biomarker data are still the norm. Thus, several patients are left behind at the moment of considering targeted therapies for critical situations. Therefore, validation of actionable pathways of disease in COVID-19 and identification of subphenotypes based on biomarker/multimodal data could offer new opportunities to reapproach targeted medicine. This unbiassed approach could potentially assist in providing the most benefit to our patients with the least harm.

## Funding Support and Author Disclosures

Dr Nadkarni has received consulting fees from AstraZeneca, Reata, BioVie, Siemens Healthineers, Variant Bio, and GLG Consulting; has received grant funding from Goldfinch Bio and Renalytix; has received financial compensation as a scientific board member and advisor to Renalytix; owns equity and stock options in Renalytix and Pensieve Health as a cofounder; and is supported by R01DK127139 and R01HL155915. Dr Vasquez-Rios has reported that he has no relationships relevant to the contents of this paper to disclose.
